# Context Specificity of the ANS Stress Response during Two Regrouping Experiments in Goats

**DOI:** 10.3389/fvets.2016.00058

**Published:** 2016-08-08

**Authors:** Antonia Patt, Lorenz Gygax, Beat Wechsler, Edna Hillmann, Jan Langbein, Nina M. Keil

**Affiliations:** ^1^Centre for Proper Housing of Ruminants and Pigs, Federal Food Safety and Veterinary Office FSVO, Agroscope, Ettenhausen, Switzerland; ^2^Ethology and Animal Welfare Unit, Institute of Agricultural Sciences, ETH Zurich, Zurich, Switzerland; ^3^Institute of Behavioural Physiology, Leibniz Institute for Farm Animal Biology, Dummerstorf, Germany

**Keywords:** heart rate, heart rate variability, goats, regrouping, stress, behavior, HPA axis

## Abstract

The aim of this study was to analyze whether the activity of the autonomic nervous system (ANS) differs between two regrouping procedures in goats, which would indicate stimulus specificity of these stressors. Applying two regrouping procedures, we evaluated heart rate and heart rate variability (RMSSD, SDNN, and RMSSD/SDNN). The two regrouping procedures were (1) introduction of individual goats into established groups (“introduction experiment”) and (2) temporary separation and subsequent reintegration of individuals from/into their group with two levels of contact during separation (“separation experiment”). In the “introduction experiment,” the heart rate of introduced goats while lying decreased continuously from an average 78 to 68 beats/min from before the introduction to the last day of the introduction period. Inversely, RMSSD increased continuously from 41 to 62 ms, which, on its own, would indicate an adaptation to the situation. During the “separation experiment,” heart rate while lying was higher when goats were separated in the “acoustic contact treatment” (82 beats/min on average) compared with the “restricted physical contact treatment” (75 beats/min on average). This difference reflected a higher level of arousal during the “acoustic contact treatment.” However, heart rate activity did not allow detecting effects of separation or reintegration. Even though it can be assumed that both the separation and introduction of goats are stressful for goats, the ANS reactions observed in this study differed between the two management procedures indicating that the ANS activation was specific to each situation. In addition, we discuss the ANS results in context with earlier findings of variables of the hypothalamic pituitary adrenal (HPA) axis (fecal cortisol metabolites) and behavior (lying and feeding). As correspondence between ANS, HPA, and behavioral reactions was limited both within and across experiments, the results of this study underline the concept that stress response patterns are context specific.

## Introduction

The physiological stress response is an important variable that can help to assess animal welfare (1, 2). Together with the hypothalamic pituitary adrenal (HPA) axis (3), the autonomic nervous system (ANS) (2) is one of the key regulatory systems of the physiological stress response. The ANS has two branches: the sympathetic nervous system and the parasympathetic nervous system. These two systems interact, have complementary roles and usually work in an antagonistic fashion: whereas the sympathetic nervous system predominates during “fight and flight” situations, the parasympathetic nervous system predominates during resting conditions (4). One commonly applied way of identifying both, sympathetic- and parasympathetic-mediated changes in the activity of the ANS is by measuring heart rate and heart rate variability (5–10). Generally, the balance between the sympathetic and the parasympathetic branch of the ANS is assessed, and a decrease in parasympathetic activity is associated with a stress response that might or might not be additionally reflected in an increased heart rate (2).

However, findings are not straightforward. For example, in small ruminants, there are three studies comparing ANS responses across stressful situations: an increase in heart rate and a decrease in heart rate variability (root mean square of successive differences of interbeat intervals, RMSSD; milliseconds) were measured when goats were forced to feed closer than they would freely choose and suggest a decrease in parasympathetic activity for lower ranking goats. However, when the same individuals were shortly isolated from their groups, heart rate increased, whereas heart rate variability (RMSSD) remained unchanged, indicating that the sympathetic branch of the ANS was activated (5). Furthermore, Désiré et al. (11, 12) showed that, in lambs, the sudden appearance of an object was characterized by a startle response and an increase in heart rate most likely due to enhanced sympathetic activity. On the other hand, the exposure to a novel object was characterized by an orientation response, an increase in heart rate variability (RMSSD), and unchanged heart rate. Taken together, there is evidence that the specific context of a situation (qualitative aspect of a stressor) may be reflected in the ANS response pattern, and this evidence needs to be tested in further studies. Additionally, such an approach should be extended to comparisons between different regulatory systems, e.g., comparing ANS and the HPA axis (8, 13–17).

Dairy goats are commonly introduced into new groups in order to restock the herd or increase its size or they are separated from familiar groups for short periods of time. Both the introduction of unfamiliar animals into an established group and the separation from the group were found to be associated with negative effects on welfare based on behavioral variables and HPA axis activity (18–22). The introduction into an established group can be assumed to have stronger negative effects on the welfare of goats than a separation as introduced goats always have to deal with both the separation from their original group and the effects of being confronted with unfamiliar conspecifics. Here, we used these two socially stressful regrouping procedures to investigate whether we would obtain similar or different ANS response patterns. In the “introduction experiment,” individual goats were introduced into unfamiliar established groups for 5 days. During the “separation experiment,” individuals were temporarily separated from and subsequently reintegrated into their original groups with two different levels of contact during separation (“acoustic contact” and “restricted physical contact”).

In accordance with previous results on behavior (lying and feeding) and HPA axis activity [fecal cortisol metabolites; Ref. (23, 24)], we expected both regrouping situations to be perceived as aversive by the goats. In particular, we anticipated a decrease in RMSSD and an elevated heart rate in the “introduction experiment” indicating that the welfare of the individually introduced goats was negatively affected. Similarly, we expected the separation and, to a smaller extent, the subsequent reintegration to be associated with a decrease in RMSSD and an increased heart rate. Additionally, we anticipated that these effects would be stronger when only acoustic contact was allowed during separation compared with additional visual and tactile contact. Furthermore, the ANS response was expected to be stronger for the introduction than the separation.

## Materials and Methods

### Animals and Housing Conditions

In both the “introduction experiment” and the “separation experiment,” goats had been grouped at least 2 months prior to the studies. Individuals of various Swiss milking breeds (Saanen, Toggenburger, Appenzeller, Chamois, Colored, St. Gallen Booted, Grisons Striped, Peacock, and Valais Blackneck) and their crossbreeds were used. We were prepared to terminate the experiments if a goat was attacked with a high risk of being injured, showed more than very mild injuries (such as abrasions or small circumscribed subdermal hematoma), or showed any other sign of illness (e.g., clinical signs of ketosis). The decision to stop an experiment with a specific animal could have been made during the periods of direct observation, fecal sampling, while heart rate measurement equipment was attached, or during the times the goats were fed. Throughout the two experiments, termination was never required. Ethical approval was obtained from the Cantonal Veterinary Office, Thurgau, Switzerland (Approval No. F4/09).

In both the “introduction experiment” and the “separation experiment,” groups were housed in the same building in identical pens allowing for acoustic and visual contact. The total area of each pen was 15.3 m^2^ (approximately 3 m × 5 m), consisting of a deep-bedded straw area and an elevated feeding place divided by a wooden wall into two equal sized compartments. The deep-bedded area was further structured by a wooden platform and a freestanding partition providing climbing opportunities as well as elevated lying areas above and protected lying areas below the platform. Hay was provided *ad libitum* in the feeding area from a 3-m long hayrack refilled twice daily at around 0845 hours and 1700 hours. One water trough, one licking stone and a brush were provided in each pen. For more detailed descriptions of housing conditions, see Patt et al. (23, 24). During both experiments data on heart rate activity, HPA axis activity (fecal cortisol metabolites) and behavior (lying and feeding duration) were recorded in the same goats.

#### Introduction of Individual Goats into Small Established Groups (“Introduction Experiment”)

In the “introduction experiment” (conducted from November 2009 to January 2010), four groups, each consisting of six adult, female, non-lactating goats, were included. Two of these four groups were composed of horned and two of hornless goats. Four further groups of six goats (adult, female, non-lactating goats, two groups horned and two hornless) provided the animals to be introduced.

#### Temporary Separation and Subsequent Reintegration of Individual Goats (“Separation Experiment”)

The “separation experiment” was conducted from March to July 2010 utilizing four experimental groups, each consisting of seven horned, adult, female, non-lactating goats. During the separation period, a pen for a single goat was set up in the home pen, reducing the space for the remaining six goats to 11.8 m^2^, but keeping space per animal constant. The pen within the home pen measured 3.5 m^2^ and served as separation pen for a treatment with restricted contact. In this treatment, the pen partition allowed restricted physical contact, i.e., visual, acoustic, and tactile contact through bars with group members. The separation for the acoustic contact treatment consisted of a lying hutch outside the barn with a deep-bedded straw area of 2.4 m^2^ and a 1.1 m^2^ outdoor area and allowed only acoustic contact to the group. Since two of the four separated goats per experimental period were in the acoustic contact treatment, two of these pens were used at the same time. The two pens were adjacent to each other and allowed visual, acoustic, and tactile contact between two unfamiliar goats through bars.

### Experimental Procedures

#### Introduction of Individual Goats into Small Established Groups (“Introduction Experiment”)

In total, 16 different goats were introduced into the four experimental groups, that is, four subsequent introductions took place in each of the groups. Half of the introductions (*n* = 8) involved horned goats, the other half hornless goats. Horned and hornless goats were introduced only into groups of goats with the same (i.e., their own) horn status. Goats of all three rank categories (see Dominance Relationships) were introduced. ANS measurements were taken during two periods: a reference (day -3) and an introduction period (days 0, 2, and 4). After the 5 days of introduction, introduced goats were brought back into their original groups. No experimental manipulations were performed during the reference period. Thus, measurements taken during the reference period served as a control to which data collected during the introduction period were compared.

#### Temporary Separation and Subsequent Reintegration of Individual Goats (“Separation Experiment”)

Three goats per experimental group (12 goats in total) were separated one at a time from their groups for 2 days and then brought back to the group. Each of the individually separated goats was separated twice from its group, once in the “restricted physical contact treatment” allowing for acoustic, visual, and tactile contact with her group through metal bars, and once in the “acoustic contact treatment” allowing only acoustic contact with her group. In each group, one goat of each of the three rank categories (see Dominance Relationships) was separated. The order in which goats experienced the two treatments was balanced across rank categories (across individuals of all groups). As isolation (= no contact with conspecifics) is known to be a potent stressor for gregarious animals, each goat separated in the “acoustic contact treatment” had tactile contact through metal bars with another simultaneously separated, unfamiliar goat. The experimental period consisted of three periods: a reference (day -3), a separation (day 0), and a reintegration period (days 2 and 4). No experimental manipulations were performed during the reference period. Thus, measurements taken during the reference period served as a control to which data collected during the separation and reintegration period were compared.

#### Dominance Relationships

A few days before the start of both experiments, the dominance relationships of the goats in each group were evaluated by direct observation during morning and evening feeding times according to the method used by Aschwanden et al. (25). Indicators for dominance and subordinance were being the active party in agonistic behavior and avoidance behavior, respectively. A goat was considered dominant if she forced another goat to leave her current position. For each pair of goats within a group, a clear unidirectional relationship was presumed if at least three agonistic interactions with the same goat being dominant were observed. If one of these three outcomes was contradictory (= bidirectional relationship), at least one additional agonistic interaction was observed for the pair concerned until one goat was twice as often clearly dominant over the other. With the help of a rank index [between 0 = omega and 1 = alpha; see Ref. (25) for information on the calculation of the rank index], each goat was categorized in relation to the other goats of her group as either low- (0.0–0.33), medium- (0.34–0.66), or high-ranking (0.67–1.00).

### Heart Rate and Heart Rate Variability

To measure heart rate activity non-invasively, we used the Polar Team^2^ Pro system (Polar^®^ Electro Oy, Kempele, Finland) consisting of a chest belt with two integrated electrodes, a data logger, an interface for downloading the data to a PC, and a corresponding software. To ensure that R–R data (i.e., intervals between successive heartbeats) measured with the Polar system adequately reflected changes in heart rate variability, and to detect and describe typical artifacts with Polar (26), we recorded exemplary R–R data simultaneously with an electrocardiogram (ECG, 3-channel digital Holter Lifecard CF^®^, Pathfinder 9.019, SPACELABS Healthcare, Snoqualmie, WA, USA) independent of the two described experiments in two goats. Measurements were obtained from subjects that were also used for the actual studies (goat “H” in Table 1 and goat “J” in Table 2). To assess comparability between the two recording systems, the durations (in milliseconds) of 200 successive R–R intervals were compared for each goat using a Spearman’s correlation test [using JMP^®^ version 12.0.1; Ref. (27)]. Results showed that measurements corresponded well between the two systems for periods when the animals were lying with correlation coefficients of 0.998 and *p*-values of <0.0001 for both goat “H” and “J.” Consequently, comparisons between studies using different devices seem to be legitimate.

**Table 1:** **Number of R–R segments used per goat and day during the introduction experiment during the reference period (day -3) and introduction period (days 0, 2, and 4)**.

		Reference period	Introduction period		
Goat	Presence of horns	Day -3	Day 0	Day 2	Day 4
A^a^	Horned	7	12	12	16
B^a^	Horned	–	7	6	12
C^a^	Horned	–	6	–	–
D^a^	Horned	6	7	16	15
E	Horned	5	10	8	17
F	Horned	4	–	–	1
G	Horned	10	–	2	12
H	Horned	2	9	9	9
I	Hornless	3	12	15	15
J	Hornless	–	–	–	–
K	Hornless	12	9	12	1
L	Hornless	7	8	3	6
M	Hornless	10	8	9	14
N	Hornless	–	13	10	10
O	Hornless	10	9	10	3
P	Hornless	7	2	–	2

*^a^Goats that were measured both during the separation and the introduction experiment*.

**Table 2:** **Number of R–R segments used per goat and day during the separation experiment during the reference period (day -5), separation period (day 0), and reintegration period (days 2 and 4)**.

		Reference period	Separation period	Reintegration period	
		
Goat	Treatment	Day -5	Day 0	Day 2	Day 4
A^a^	Acoustic contact	8	5	14	12
B^a^	Acoustic contact	–	–	–	–
C^a^	Acoustic contact	–	–	–	–
D^a^	Acoustic contact	5	8	4	5
E	Acoustic contact	5	6	3	–
F	Acoustic contact	–	–	1	–
G	Acoustic contact	1	–	6	4
H	Acoustic contact	–	6	9	–
I	Acoustic contact	–	4	3	2
J	Acoustic contact	2	8	1	2
K	Acoustic contact	7	10	11	11
L	Acoustic contact	2	–	–	–
A^a^	Restricted physical contact	9	–	7	9
B^a^	Restricted physical contact	–	2	–	–
C^a^	Restricted physical contact	–	–	–	–
D^a^	Restricted physical contact	3	–	6	2
E	Restricted physical contact	–	6	5	10
F	Restricted physical contact	3	9	3	2
G	Restricted physical contact	–	–	8	1
H	Restricted physical contact	–	5	–	–
I	Restricted physical contact	1	12	9	10
J	Restricted physical contact	2	13	7	–
K	Restricted physical contact	3	11	–	12
L	Restricted physical contact	–	–	–	3

*^a^Goats that were measured both during the separation and the introduction experiment*.

In all introduced (“introduction experiment,” between-subject design: *n* = 16 goats) and separated (“separation experiment,” within-subject design: *n* = 12 goats × 2 treatments) goats, heart rate activity was measured. Four goats took part in both experiments. For those goats, heart rate and heart rate variability were measured both in the “introduction experiment” and the “separation experiment” (Tables 1 and 2). When equipping goats with the chest belt, one electrode was placed on the thoracic wall directly behind the left olecranon and the other in the distance given by the chest belt on the left thoracic wall. To increase the electrode–skin contact, the spots of electrode application were depilated (Veet^®^ hair removal cream, Reckitt Benckiser Switzerland AG, Wallisellen-Zurich, Switzerland) and electrode gel (Electrode cream, Anandic Medical Systems AG/SA, Diessenhofen, Switzerland) was used. Furthermore, to improve the initial electrode–skin contact, two water-soaked sponges were attached to the chest belt on the level of the two electrodes. The chest belt was protected by an elastic stretch belt.

In both experiments, data were collected on a reference day (“introduction experiment”: day -3; “separation experiment”: day -5) and three times (days 0, 2, and 4) during the following regrouping treatment. For the “separation experiment,” this included both the separation period (day 0) and the reintegration period (days 2 and 4). As daily routines did not vary between days -7 and -1 during the reference period of both the “introduction” and “separation experiment,” the choice of the specific reference day is unlikely to affect outcome measures and depended on other measures that were taken during the reference period. To study the longer-term effects of these two regrouping procedures, it is necessary to minimize influences caused by different levels of physical activity (9). Thus, to choose times that clearly reflected resting period, we considered only data obtained during lying periods at night which lasted at least 30 min. On each observation day, goats were fitted with the device around 2030 hours, and the device was removed the next morning around 0600 hours. Lying behavior was recorded by using a commercial 3D acceleration logger (MSR145WA, Modular Signal Recorder Electronics GmbH, Seuzach, Switzerland; 33 mm × 15 mm × 61 mm) attached to the left hind legs as described in Patt et al. (23, 24). Given the 30-min selection criterion, it is possible that goats were sleeping during some of the selected lying periods. In several species, higher RMSSD measures have been observed during the night or while sleeping, which is assumed to be due to higher parasympathetic activity (28, 29). If our data sets included heart rate measures while goats were either sleeping or not, variability in our heart rate activity measures would have increased. Nevertheless, we found systematic changes in these variables, specifically in the “introduction experiment” where one could have expected that such variability had a higher influence because of the between-subject design (see above) compared with the “separation experiment” in which we used a within-subject design (see above). Additionally, the variability in the heart rate activity measures did not differ between the two experiments.

The automatic correction of the tachogram was done with the Polar Equine SW4 software (Polar^®^ Electro Oy, Kempele, Finland). Different approaches exist for the analysis of heart rate variability. Time domain-related measures that are based on differences in variability between inter beat intervals, i.e., variability over time, are most commonly used. Additionally to time-related measures, spectral measures, i.e., measures based on differences between the high- and low-frequency components of the heart rate variability spectrum, are widely applied. We chose time domain-related measures over spectral measures as RMSSD (root mean square of successive differences of interbeat intervals; milliseconds) and SDNN (SD of all interbeat intervals; milliseconds) are highly correlated with the spectral measures high frequency (HF) band and low frequency (LF) band, respectively (10, 30), and are more easily interpretable than some of the spectral measures. For each period of 30 min of uninterrupted lying, a 5-min segment of the tachogram, neither at the beginning nor at the end of the 30-min period and with a corrected fault rate of <5%, was included in the analysis. Consequently, we chose to be conservative in our choice of tachogram segments as in goats data with a 10% fault rate have been found to be acceptable and published (9). Based on these 5-min segments, we calculated [in R (31)] heart rate (beats/minute), RMSSD as a variable of vagal activation (2), and SDNN as a variable of sympathetic and/or vagal activation (2). Additionally, the ratio between RMSSD and SDNN was calculated as a variable of changes in the vagal-sympathetic balance (32).

Data included in the analysis of the introduction and separation experiment are summarized in Tables 1 and 2, respectively, and resulted in a somewhat unbalanced data set (see Statistical Analysis). In both experiments, two causes for missing data occurred, either no valid signal was detected during the selected periods, for example, when electrodes slipped from their original position, or the tachograms of the available 5-min segments had a fault rate of more than 5% and were thus excluded from the analysis.

### Statistical Analysis

To adequately reflect dependencies in the experimental design (nesting, repeated measurements), linear mixed-effects models were used to evaluate the outcome variables. Statistical analysis was performed in R (31) by using the lmer method from the lme4 package (33) as well as the function dredge from the MuMIn package (34) to perform all-subset analyses. Model estimation was therefore base on a maximum-likelihood approach. This approach can correctly deal with unbalanced data sets (see Heart Rate and Heart Rate Variability), that is, each goat/observation contributes in estimating the absolute level and the relative differences between experimental conditions in an outcome variable specific to the extent of data availability. A single data point of an animal hardly contributes in the estimation at all, whereas two data points and more contribute to estimating the relative differences between the available situations.

For both experiments, outcome variables were heart rate (beats/minute), RMSSD (milliseconds), SDNN (milliseconds), and RMSSD/SDNN. Outcome variables were log transformed, and models were calculated separately for both experiments. Random effects were *date* nested in *animal* nested in *housing group* for the “introduction experiment” and *date* nested in *treatment* nested in *animal* nested in *housing group* for the “separation experiment.”

For the “introduction experiment,” fixed effects were *day* (factor with four levels: days -3, 0, 2, and 4), *presence of horns* (factor with two levels: yes, no), and *rank category* (factor with three levels: high-, medium-, or low-ranking). For the “separation experiment,” fixed effects were *day* (factor with four levels: days -5, 0, 2, and 4), *treatment* (factor with two levels: acoustic contact, restricted physical contact), and *rank category* (factor with three levels: high-, medium-, or low-ranking). Testing for effects of *day* allowed us to detect if outcomes varied throughout the experiment. The relevance of these changes can be assessed by comparing effect sizes across days.

An all-subset analysis was conducted for each outcome variable in both experiments, ranging from the minimal model including a constant (intercept) only (35, 36) to the model including the three fixed effects and all two-way interactions in the “introduction experiment,” and the three fixed effects and all their interactions in the “separation experiment.” The model including a constant corresponds to the null hypothesis that no explanatory variable has an influence and that the responses vary randomly around a general mean. In the “introduction experiment,” the model with all possible two-way interactions was the maximum model because the models were over specified when they included the three-way interaction due to missing values of the combination of medium-ranking horned goats on the reference day (day -3). Thus, the total number of models analyzed for each outcome variable was 64 in the “introduction experiment” and 128 in the “separation experiment” (sets of models). The choice among the different models was based on Akaike’s information criterion corrected further for small sample sizes (AIC_c_), and on the Akaike weight (*w*_i_). For each outcome variable, the Akaike weights (*w*_i_) of all models in the set add up to one. Thus, the Akaike weight (*w*_i_) can be interpreted as the probability of a given model to fit the data best within the set of models (35, 37, 38). For all outcome variables, the chosen model based on the Akaike weight (*w*_i_) is shown in Table 3. If models with a similar model probability were nested (i.e., the factors of the simpler model were included in the more complex models) and had similar AIC_c_ values, we chose the simpler model (39). This implies that factors only included in the more complex models are thought to have only marginal importance. It is indicated in the results whenever the simpler model instead of that with the highest probability was chosen, and the model’s probability in comparison with the most probable model is given. Model selection is thus based on the models’ relative fit within the given data set. To substantiate the relative strength of the chosen model within the set, we also report the evidence ratio of the chosen model in comparison with the null model (ER_0_), providing a measure of how much more likely the chosen model is than the null model (38). This statistical approach takes into account that any model is only an approximation of the hypothesis investigated. Single fixed effects are no longer significant (or not), but the chosen model as a whole represents the approximation that best explains the observed data. This approach of model selection is conceptually different to the classical step-wise backwards testing, as it tests the probability of a specific model given the data (35, 38). In analogy to a classical frequentist approach, effect sizes of the fixed effects need to be considered to assess biological relevance (38).

**Table 3:** **Models selected for describing the effects of heart rate variables based on AIC_c_ for results obtained in an “introduction experiment” and a “separation experiment” with goats**.

Outcome variable	Selected models^a^	*w*_i_^b^	ER_0_^c^
**Introduction experiment**			
Heart rate (beats/minute)	Day	0.41	10.3
RMSSD (ms)	Day	0.20	5.0
SDNN (ms)	Day	0.28	140.0
RMSSD/SDNN	Intercept	0.16	1.0
**Separation experiment**			
Heart rate (beats/minute)	Treatment	0.19	3.8
RMSSD (ms)	Intercept	0.42	1.0
SDNN (ms)	Intercept	0.34	1.0
RMSSD/SDNN	Intercept	0.46	1.0

*RMSSD, root mean square of successive differences of interbeat intervals, milliseconds; SDNN, SD of all interbeat intervals, milliseconds; RMSSD/SDNN, ratio between RMSSD and SDNN*.

*^a^Fixed effects included in the model chosen by Akaike’s information criterion (AIC_c_)*.

*^b^w_i_ = Akaike weight, which can be interpreted as the probability of the given model within the set*.

*^c^ER_0_ = Evidence ratio between the chosen model and the null model (including only the intercept)*.

## Results

### Introduction Experiment

**Figure 1:**
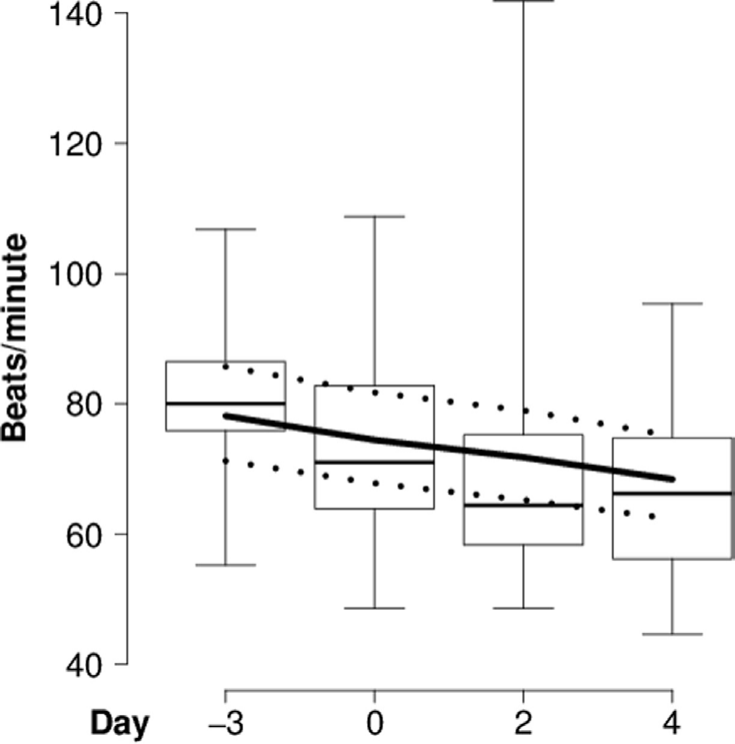
**Heart rate of introduced goats**. Mean heart rate (beats/minute for each day) of individually introduced goats during lying in the “introduction experiment” before (day -3) and during (days 0, 2, and 4) the introduction period. Box-and-whisker plot: boxes = first and third quartile, thick line = median, whiskers = range from minimum to maximum value. Solid lines = model estimates, dotted lines = 95% confidence intervals.

**Figure 2:**
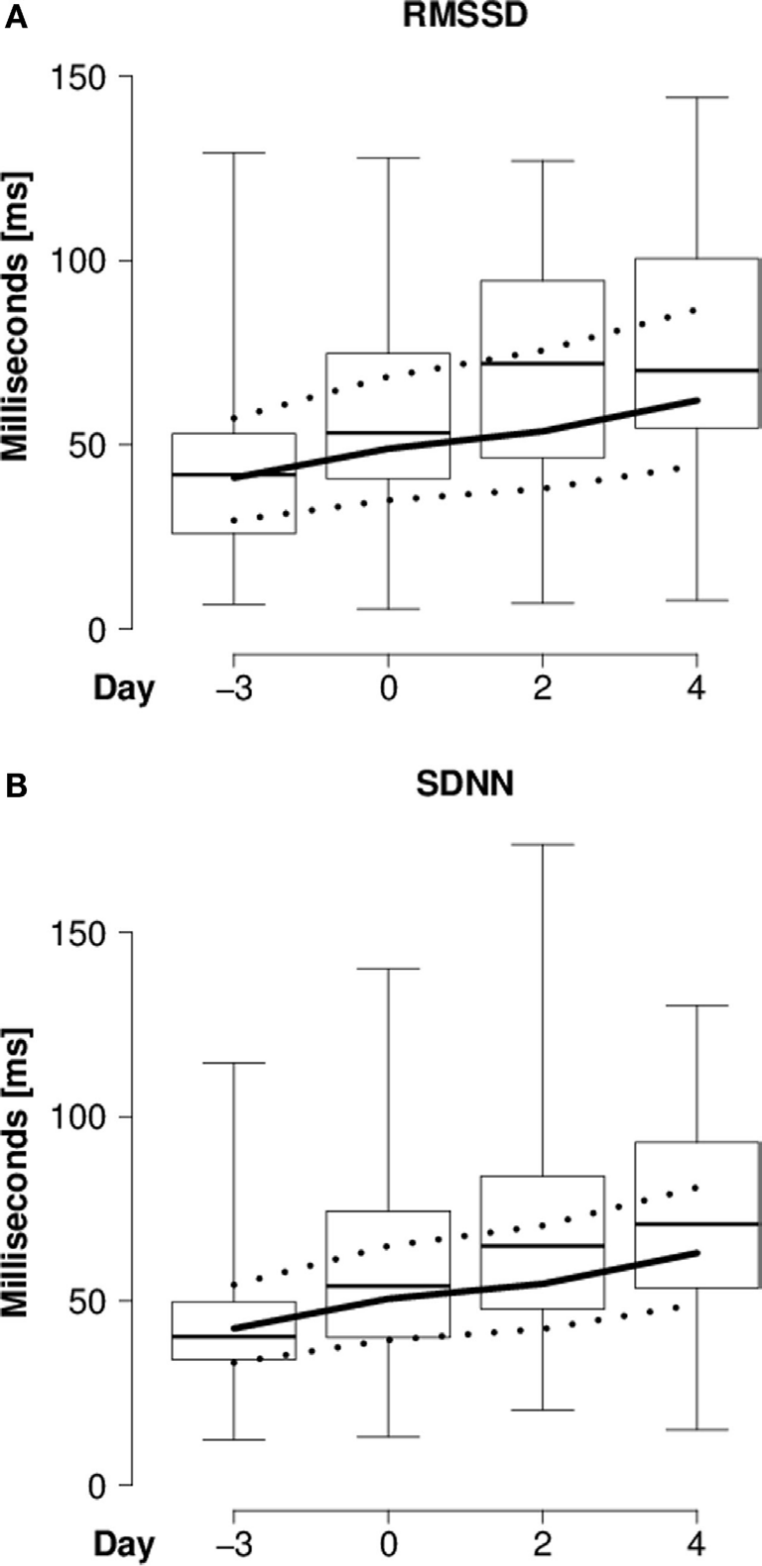
**Heart rate variability of introduced goats**. **(A)** RMSSD (milliseconds) and **(B)** SDNN (milliseconds) of individually introduced goats during lying in the “introduction experiment” before (day -3) and during (days 0, 2, and 4) the introduction period. Box-and-whisker plot: see Figure 1.

In the “introduction experiment,” heart rate continuously decreased (Table 3; Figure 1), whereas RMSSD and SDNN continuously increased in the course of the introduction period compared with the reference day (Table 3; Figures 2A,B). For both RMSSD and SDNN, these were the models with the second highest probability [their probability and evidence ratio being 0.83 and 0.97, respectively, in comparison with the models with the highest probability which additionally to *day* included *presence of horns* and *rank category* (RMSSD) or *presence of horns* (SDNN) as main effects]. For RMSSD/SDNN, the null model, which was the model with the third highest probability (with a probability of 0.48 in comparison with the most probable model which included *rank category* as a main effect), was chosen.

### Separation Experiment

**Figure 3:**
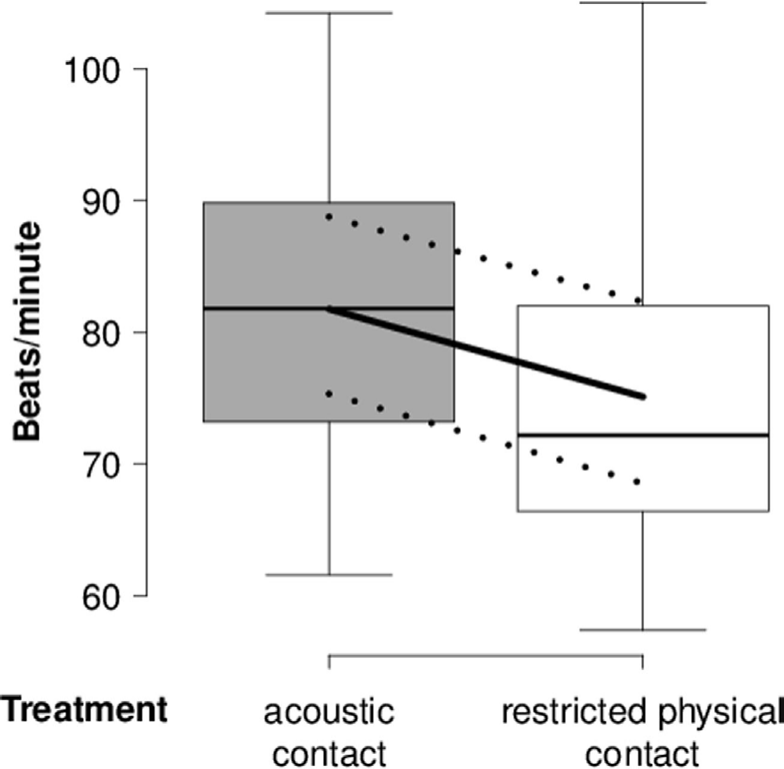
**Heart rate of separated goats**. Mean heart rate (beats/minute) of separated goats during lying in the “separation experiment” in relation to the “acoustic contact” and “restricted physical contact treatment.” Box-and-whisker plot: see Figure 1.

In the “separation experiment,” heart rate was lower during the “restricted physical contact treatment” than the “acoustic contact treatment” (Table 3; Figure 3). The null model was the model with the highest probability for RMSSD, SDNN, and RMSSD/SDNN. None of the three outcome variables were detectably affected by *treatment, day*, or *rank category*.

## Discussion

In this study, we measured the effects of two different regrouping procedures (“introduction” and “separation”) on the regrouped goats’ heart rate and heart rate variability to assess context specificity of the two social stressors. The stress response of the ANS was clearly different in the two regrouping procedures and supports our hypothesis that the qualitative aspect of a stressor is reflected in the ANS response pattern: in the “introduction experiment,” heart rate decreased and heart rate variability increased in the course of the introduction period. In the “separation experiment,” heart rate was higher during the “acoustic contact treatment” than the “restricted physical contact treatment.” However, heart rate activity did not indicate an effect of the separation or reintegration *per se* (no day-to-day effect). Surprisingly, the patterns of heart rate activity data seem to contradict the patterns of behavioral data and fecal cortisol metabolites concentrations recorded in the same animals at first sight (23, 24). Although it was concluded previously (23, 24) that both regrouping procedures negatively affected the goats’ welfare, the current ANS results show an increased parasympathetic activation in the “introduction experiment” and no visible change in ANS reaction in the “separation experiment.”

### Context Specificity of Heart Rate and Heart Rate Variability

In the “introduction experiment,” the continuously decreasing heart rate of the introduced goats and the simultaneously increasing RMSSD can be interpreted as an increasing activity of the parasympathetic branch of the ANS, indicating an increasing adaptation to the situation. This assumption is not contradicted by our results regarding SDNN, which reflects mixed sympathetic and parasympathetic activity (2). If the increasing SDNN values observed in the present experiment reflected increasing sympathetic activity, heart rate would have been expected to remain more or less constant (11, 12), as SDNN increased to a similar extent as RMSSD.

For the “separation experiment,” the generally higher heart rate of lying goats throughout the “acoustic contact treatment” compared with that of goats in the “restricted physical contact treatment” reflected a higher level of arousal. Since this difference did include the reference day, the difference is difficult to explain because group effects were accounted for by the random effect and sequential effects by the balancing of the order of the treatments. Regarding heart rate variability, neither RMSSD nor SDNN was affected by *treatment, day*, or *rank category*. Compared with reference measures, RMSSD did not change considerably during short-term separation of goats in a previous study, either (5). Thus, results suggest that short-term separation has no longer-term effects on the activity of the parasympathetic nervous system.

The different ANS response in the two regrouping procedures suggested that the two situations were perceived differently by the animals. This approach of comparing response patterns of the ANS between situations (5, 11, 12) should be extended in further research by systematically modifying quantitative and qualitative characteristics of a stressor to see whether this is reflected in distinct response patterns. At best, this approach would also put heart rate activity data in context with other variables and especially integrate different regulatory systems, i.e., the patterns of effects of variables of the ANS and the HPA axis.

Although the regulating centers of the HPA axis and the ANS are interconnected, and, e.g., the hypothalamic paraventricular nucleus (PVN) appears to be a major center of autonomic and neuroendocrine integration (40–42), the activation of each of the two regulatory systems is not uniform but differs depending on the characteristics of the stimulus. Whereas it has repeatedly been shown that qualitative aspects, such as controllability and predictability, play a role in the activation of the HPA axis, far less is known about the quality of stimuli that activate the ANS. In sheep, heart rate activity patterns differ depending on stimulus characteristics such as suddenness and unfamiliarity (11, 12). Human research suggests that the extent of the HPA axis activation and the cardiovascular response correlate well during very high level of stress, but not during mild or moderate stress (43). In the few available studies, measuring the effects of a specific stressor quality on both HPA axis activity and heart rate activity in farm animal species results are not straightforward. Although, in some studies, patterns of both systems led to similar conclusions regarding the welfare state (8, 13), in other studies, heart rate activity and HPA axis activity would not have allowed to draw the same conclusions on their own (14–17). These differences could depend on the specific function of the two systems, whereas physical activity affects heart rate and the heart rate variability patterns (2), the main function of the HPA axis is the regulation of energy distribution, i.e., its activation can be due to metabolic mobilization, without necessarily a correlation to perceived stress (1).

### Comparing ANS Reactions to Behavior and HPA Axis Activation during Introduction and Separation

To see whether indicators of the ANS, HPA axis, and behavior would lead to the same conclusions regarding the effects of two regrouping procedures on goats, we compared the measures of heart rate activity with other etho-physiological measures. The other measures were recorded simultaneously during the same two regrouping experiments and have already been published [Ref. (23, 24); Supplementary Material]. Taking into account all variables measured, it seems that a selection of the variables, that is, for example, only behavioral variables or only variables of the HPA axis or heart rate activity, would have led to different interpretations of the effects of these two situations on animal welfare. For the “introduction experiment,” common interpretation of the heart rate activity data would suggest that introduced goats increasingly adapted to the situation over time, that is, they became increasingly calmer throughout the integration. However, since values rose starting from the reference values, did not stabilize or shift back to values observed during the reference period, it is doubtful whether the RMSSD pattern is really indicative of progressive adaptation. Furthermore, the increase in and consistent high level of cortisol metabolites concentrations during introduction contradict an adaptation process and, together with behavioral data (considerably increased lying and decreased feeding duration), indicate lasting negative consequences of introducing goats individually into small established groups (23). For the “separation experiment,” interpretation of cardiac activity alone would have led to an underestimation of the negative effect the separation had on the goats. However, higher levels of fecal cortisol metabolites concentrations and reduced lying and feeding durations allowed us to conclude that goats were negatively affected by the separation and more so than by the subsequent reintegration (24).

Compared with the ”introduced goats,” that were both separated from their group and had to deal with the challenges of being introduced into a new group, the latter was missing for “separated goats.” Therefore, they might have engaged in a more active response pattern aiming at restoring social contact (22), which was physiologically further characterized by the observed activation of the HPA axis. Considering all variables assessed in the “introduction experiment” (behavior, cortisol metabolites, and cardiac activity), there seem to be two possible explanations for the activation of the parasympathetic branch of the ANS during the introduction period and a simultaneous activation of the HPA axis: increasing frustration or fear-related bradycardia. During extinction learning in rats (44, 45) and visual discrimination learning in dwarf goats (9), reduced heart rate and increased RMSSD have been hypothesized to be associated with frustration. However, other studies in horses, sheep, and dwarf goats did not find such an effect (14, 46, 47). Taking into account the introduced goats’ exceedingly long lying durations, the observed patterns of cardiac and HPA axis activity could also reflect a kind of “freezing” reaction which can be associated with the so-called “fear bradycardia” (48, 49) that has been linked to parasympathetic activation in humans (49). It has been suggested that freezing occurs in situations in which animals have no clear information about how to act (50) or in situations from which they cannot escape (51). However, most previous studies on frustration and all studies on fear bradycardia have focused on acute effects, whereas data of this study were collected to assess longer-term effects on heart rate variables when the goats were lying.

## Conclusion

Patterns observed in heart rate and heart rate activity measured in our study differed between the two regrouping procedures support the concept of the ANS responses being context specific. Furthermore, when comparing the results of heart rate activity with those of the previously published behavioral variables and fecal cortisol metabolites, the extent of the welfare effects of separating and reintegrating goats would have been assessed differently when only a selection of variables had been taken into account. In the “introduction experiment,” the results of heart rate activity might be explained by frustration and/or fear bradycardia. This underlines the need to further investigate which characteristics of a stressor result in which behavioral and physiological response patterns. So far, this line of research has received only little attention in animal welfare research.

## Author Contributions

Study design: AP, LG, BW, EH, and NK. Data collection: AP. Data analysis: AP, LG, and JL. Manuscript drafting: AP and NK. Critical revision of the manuscript: LG, BW, EH, and JL. Final approval: AP, LG, BW, EH, JL, and NK.

## Conflict of Interest Statement

The authors declare that the research was conducted in the absence of any commercial or financial relationships that could be construed as a potential conflict of interest. The reviewers KO and JD and handling editor declared their shared affiliation, and the handling editor states that the process nevertheless met the standards of a fair and objective review.
