# Supplementary material for: Context Specificity of the ANS Stress Response during Two Regrouping Experiments in Goats
Source: Front Vet Sci. 2016 Aug 8;3:58. doi: 10.3389/fvets.2016.00058 (PMC4976294; doi:10.3389/fvets.2016.00058)
Supplement: Supplementary file 1 [file table_1.pdf]

Supplementary Material

Context specificity of the ANS stress response during two regrouping experiments in goats

Antonia Patt[1,2], Lorenz Gygax[1], Beat Wechsler[1], Edna Hillmann[2*], Jan Langbein[3], Nina M. Keil[1]

[1] Centre for Proper Housing of Ruminants and Pigs, Federal Food Safety and Veterinary Office FSVO, Agroscope, Ettenhausen, Switzerland
[2] Ethology and Animal Welfare Unit, Institute of Agricultural Sciences, ETH Zurich, Zurich, Switzerland
[3] Institute of Behavioural Physiology, Leibniz Institute for Farm Animal Biology, Dummerstorf, Germany

* Correspondence: Dr. Edna Hillmann, Ethology and Animal Welfare Unit, Institute of Agricultural Sciences, ETH Zurich, Universitätstrasse 2, 8092 Zurich, Switzerland, edna-hillmann@ethz.ch

Differences between the model selection approaches used for the previous analysis of behavioral variables and HPA-axis activity data and the current analysis of HR/HRV data

Model selection done earlier for behavior and HPA-axis activity (23, 24) was based on a smaller number of models that were set up in advance, focusing on the anticipated interactions between the fixed effects. In doing so, the kind and number of interactions included varied between outcome variables, but each model included at least all fixed effects as main effects. From those original analyses, we had therefore only weak indications of the relative statistical importance of the individual main effects. The updated model selection presented here followed the same approach as was used for the heart rate activity data (as presented in the main paper), we also used an all-subset approach: starting with the global model (i.e. including interactions between the fixed effects *day, presence of horns* and *rank category* for the "introduction experiment" and *day*, *treatment* and *rank category* for the separation experiment). Contrary to the previous model selection (23, 24), reduced models do not necessarily include all fixed effects as main effects and the smallest model possible is the null model, including only the intercept. This approach reflects the relative statistical importance of the individual fixed effects included in the analysis more clearly. The reduced models of the current analysis are shown in Supplementary Table 1. In the comparison between heart rate activity data and behavioral and fecal cortisol metabolites concentrations we focused on whether the fixed effect *day* was included in the chosen model since this allowed us to detect if outcomes varied throughout the experiment. For all outcome variables in the "introduction experiment,", that is, lying duration, feeding duration and concentration of fecal cortisol metabolites, the chosen model included *day* either as a main effect (feeding duration, fecal cortisol metabolites) or in interaction with the other fixed effects (lying duration). In the "separation experiment," the fixed effect *day* was included as a main effect in the model of feeding duration and cortisol metabolites concentrations. Further, *day* was included in a two-way interaction with treatment in the model of lying duration.

Supplementary Table 1: Models selected for describing the effects of behavior and HPA-axis activity based on AIC_c for outcomes obtained in an “introduction experiment” and a “separation experiment” with goats.

**Outcome variable**	**Selected models**[1]	w_i[2]	ER_0[3]
**Introduction experiment**			
Lying (hours/day)	*Day × Presence of horns*	0.28	> 560.0
Feeding (hours/day)	*Day + Presence of horns*	0.18	> 360.0
Fecal cortisol metabolites (ng/g feces)	*Day + Presence of horns × Rank*	0.99	> 1980.0
**Separation experiment**			
Lying (hours/day)	*Treatment × Day*	0.89	> 1780.0
Feeding (hours/day)	*Treatment × Rank + Day*	0.14	> 280.0
Fecal cortisol metabolites (ng/g feces)	*Day*	0.27	> 540.0

[1] Fixed effects included in the model chosen by Akaike’s information criterion (AIC_c)
[2] w_i = Akaike weight, which can be interpreted as the probability of the given model within the set
[3] ER_0 = Evidence ratio between the chosen model and the null model (including only the intercept)

Results of the updated model selection for the behavioral variables and HPA axis activity

Overall, the models resulting from the current model selection strategy for behavior and HPA-axis activity were very similar to the original models (23, 24), which indicates that the effects were robust. In summary, the effects of the introduction of individual goats into established groups were more pronounced in horned goats compared with hornless goats as indicated by substantially longer lying times (increase from about 10 hours to more than 20 hours per day in horned and to 13 hours in hornless goats) and shorter feeding times (reduction from about 4 hours to approximately 1 hour per day in horned and to 3 hours in hornless goats). That is, especially horned goats spent the majority of time lying in the niche below the wooden platform which was interpreted as the introduced goats’ attempt to hide from resident goats and to minimize agonistic social interactions. Further, concentrations of fecal cortisol metabolites were elevated about 1.5 times during the entire introduction period compared with the reference days, with highest values measured in horned, high-ranking goats. During the separation period the feeding duration of separated goats decreased from about 4 hours to around 3 hours independently of the applied treatment. Additionally, concentrations of cortisol metabolites increased on the second day of the separation period and the first day of the reintegration period (roughly by a factor of 1.5 compared with reference days). Further, in the treatment allowing for only acoustic contact (“acoustic contact only treatment”) but not in the treatment allowing for tactile, visual and acoustic contact (“restricted physical contact treatment”), lying duration was reduced from about 13 hours per day to around 9 hours on the first day of the separation period compared with reference days (see also 23, 24).
